# Genomic Data Mining Reveals Abundant Uncharacterized Transporters in *Coccidioides immitis* and *Coccidioides posadasii*

**DOI:** 10.3390/jof8101064

**Published:** 2022-10-10

**Authors:** Hong Cai, Hao Zhang, Daniel H. Guo, Yufeng Wang, Jianying Gu

**Affiliations:** 1 Department of Molecular Microbiology and Immunology, University of Texas at San Antonio, San Antonio, TX 78249, USA; 2 South Texas Center for Emerging Infectious Diseases, University of Texas at San Antonio, San Antonio, TX 78249, USA; 3 Strake Jesuit College Preparatory, Houston, TX 77036, USA; 4 Department of Biology, College of Staten Island, City University of New York, Staten Island, New York, NY 10314, USA

**Keywords:** coccidioidomycosis, *Coccidioides*, transporters, genomics

## Abstract

*Coccidioides immitis* and *Coccidioides posadasii* are causative agents of coccidioidomycosis, commonly known as Valley Fever. The increasing Valley Fever cases in the past decades, the expansion of endemic regions, and the rising azole drug-resistant strains have underscored an urgent need for a better understanding of *Coccidioides* biology and new antifungal strategies. Transporters play essential roles in pathogen survival, growth, infection, and adaptation, and are considered as potential drug targets. However, the composition and roles of transport machinery in *Coccidioides* remain largely unknown. In this study, genomic data mining revealed an abundant, uncharacterized repertoire of transporters in *Coccidioides* genomes. The catalog included 1288 and 1235 transporter homologs in *C. immitis* and *C. posadasii*, respectively. They were further annotated to class, subclass, family, subfamily and range of substrates based on the Transport Classification (TC) system. They may play diverse roles in nutrient uptake, metabolite secretion, ion homeostasis, drug efflux, or signaling. This study represents an initial effort for a systems-level characterization of the transport machinery in these understudied fungal pathogens.

## 1. Introduction

Soil-dwelling dimorphic fungi, *Coccidioides immitis* and *Coccidioides posadasii*, are causative agents of coccidioidomycosis, commonly known as Valley Fever [1]. While about 60% of infected people show no or minimum symptoms, the remainder develop clinical symptoms, ranging from pneumonia to life threatening, disseminated coccidioidomycosis. There is currently no clinically available vaccine against coccidioidomycosis and treatments are based on standard antifungal therapies.

The development of new antifungal drugs targeting *Coccidioides* is urgently needed because (1) The rise of incidence of coccidioidomycosis [2]. Incidence was reported to increase by almost 800% from 2000 to 2018 in California, and in 2018 over 15,000 cases were reported to the Centers for Disease Control and Prevention of the United States [3,4]; (2) The increase in the area of endemicity. Historically endemic in the arid and semiarid areas of the southwestern USA, Mexico, Central America, and South America [5], *Coccidioides* were recently reported to expand to Utah, Oregon, and Washington state [6,7,8,9,10]; (3) The emergence of clinical isolates conferring resistance to antifungals [11,12,13].

The search for new targets against *Coccidioides* requires a better understanding of *Coccidioides* biology. Despite recent advances in epidemiology, ecology and population biology [1,14,15], the molecular mechanisms underlying fungal growth, adaptation to the host environment, pathogenesis, and virulence, remain elusive. Difficulties in *Coccidioides* research partly stem from the fundamental complexity of *Coccidioides* fungi, which have a unique life cycle, switching between saprobic and parasitic phases. They grow in the soil, cycling between saprobic mycelia and arthroconidia. When the soil is disturbed, the arthroconidia can become airborne and be inhaled by a host. The parasitic phase is initiated when arthroconidia transform into spherules, leading to pulmonary infection.

The completion of the genome sequencing projects for *C. immitis* and *C. posadasii* provides us with an unprecedented opportunity to unveil genes and gene products that are essential for their survival and infective potential [10,16,17,18,19]. Instead of an individual-gene based approach, in this study, using genomic and systems biology approaches, we attempted to identify and catalog the transport machinery in *C. immitis* and *C. posadasii*. Transporters were chosen as the subject of this study because the complex life cycle of *Coccidioides* requires a powerful transport system. Transport is a vital function of all living cells. Transporters are an extremely diverse group of proteins that catalyze the transfer of substrates ranging from nutrients, metabolic products, macromolecules, drugs and toxins, to signaling molecules. Depending on the energy sources and translocation mechanisms, transporters can be classified as carriers, channels, pumps, electron-flow carriers, etc. The function of transporters is associated with their structural and topological features, including the presence and number of transmembrane segments. Transporters are believed to be involved in various processes in fungi, including nutrition, signaling, stress response, cellular detoxification, and virulence [20,21]. Most importantly, extensive studies have demonstrated that transporters play an essential role in multidrug resistance in pathogenic fungi [22,23,24,25,26]. In addition, the feasibility of designing inhibitors of transporters important for survival or drug resistance make them potential drug targets [26]. Despite its functional significance, the transport machinery in *Coccidioides* remains largely unexplored. Our comparative genomic analyses identified 1288 and 1235 putative transporters in *C. immitis* and *C. posadasii*, respectively. They may be involved in cellular networks regulating nutrient uptake, metabolite secretion, drug and toxin efflux, maintenance of ion content, macromolecule export, and signaling. This study represents the first step towards a systems-level elucidation of the transport machinery in the complex, yet understudied, *Coccidioides*.

## 2. Materials and Methods

### 2.1. Genomic Data

**Figure 1:**
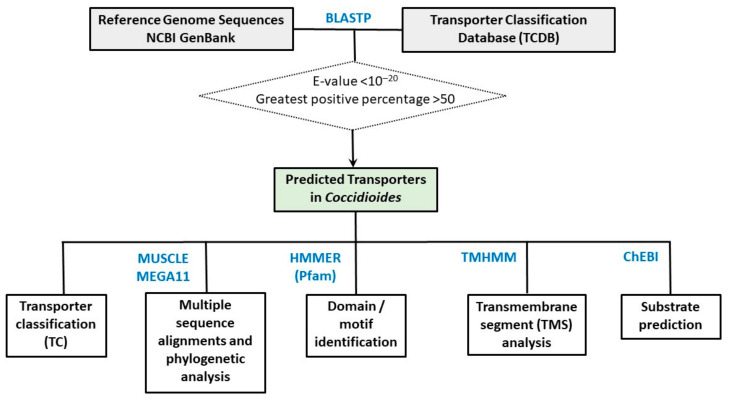
The pipeline of genomic analysis of *Coccidioides* transporters. See Materials and Methods for details.

Figure 1 depicts the pipeline of our genomic analysis. The completed reference genome sequences of *C. immitis* RS strain (BioProject PRJNA12883) and *C. posadasii* SOWgp strain (BioProject PRJNA9616) were downloaded from the NCBI Genbank database (https://www.ncbi.nlm.nih.gov/data-hub/genome/?taxon=5500, accessed on 20 July 2021) (Table 1) [16,17]. All the amino acid sequences and annotated features were retrieved.

The transporter classification database TCDB, https://www.tcdb.org/ (accessed on 20 July 2021), was used as the knowledge database for the identification and classification of transporters in *Coccidioides* [27,28]. TCDB includes curated transporter sequences, classification, structural, evolutionary, mechanistic, medical and biotechnological information about transport systems from a variety of organisms. A total of 21,373 amino acid sequences of the transporters collected in the TCDB was downloaded.

### 2.2. Identification, Classification, and Characterization of Putative Transporters in Coccidioides

The BLASTP query of all the proteins in *C. immitis* RS strain and *C. posadasii* SOWgp strain against the TCDB database was conducted to identify *Coccidioides* proteins that were homologs to known or predicted transporters [29]. The cutoff for homologous genes were set as: BLASTP E-value < 10^−20^ and greatest positive percentage >50. The annotation of *Coccidioides* transporters was based on the hits in the TCDB with the lowest E-value, and the highest similarity score.

The predicted *Coccidioides* transporters were further classified into families and subfamilies based on the 5-letter Transport Classification (TC) system [13]. Similar to the Enzyme Commission (EC) system for classification of enzymes, the TC system is formally adopted by the International Union of Biochemistry and Molecular Biology for transporter classification and nomenclature. The 5-letter TC number, in the form of VWXYZ corresponds transporter class, subclass, family, subfamily and the substrate/substrates transported [27].

Conserved domains/motifs in predicted *Coccidioides* transporter sequences were identified by searching against the Pfam 35.0 database [30], which is a collection of protein families based on hidden Markov models implemented in HMMER [31]. The TMHMM program was used to analyze the transmembrane structures and predict transmembrane segments (TMSs) [32]. The substrates for predicted *Coccidioides* transporters were predicted based on the Chemical Entities of Biological Interest (ChEBI), an ontology and dictionary focused on small chemical compounds [33].

### 2.3. Multiple Alignment and Phylogenetic Analysis

Multiple sequence alignments were obtained using the MUSCLE program [34,35]. Phylogenetic trees were inferred by the neighbor-joining methods [36] and the maximum likelihood [37] using MEGA11 [38]. Bootstrap resampling with 1000 pseudo replicates was used to assess statistical support for each branch [39].

## 3. Results

### 3.1. C. immitis and C. posadasii Possess a Rich Repertoire of Transporters

To gain insight into the transport machinery of *Coccidioides*, the protein sequences in the *C. immitis* RS strain and *C. posadasii* SOWgp strain were subjected to an exhaustive search against the TCDB database, which has a catalog and a structure-, mechanic-, and phylogeny-based classification of transporters. Stringent threshold of E-value < 10^−20^ and positive percentage >50 were adopted to ensure the high coverage with low false-positives. A total of 1288 and 1235 transporter homologs were identified in *C. immitis* and *C. posadasii*, respectively, which account for 13.0% and 17.1% of their respective proteome (Table 1). The transporter composition in the two organisms was highly similar (Supplementary Table S1): Mutual BLASTP analysis between *C. immitis* and *C. posadasii* showed that only 23 transporters were specific to *C. immitis*, and six transporters were specific to *C. posadasii*. Our new catalog of transporters included 197 and 103 transporters that were previously annotated by genome-sequencing projects in *C. immitis* and *C. posadasii*, respectively [17].

**Figure 2:**
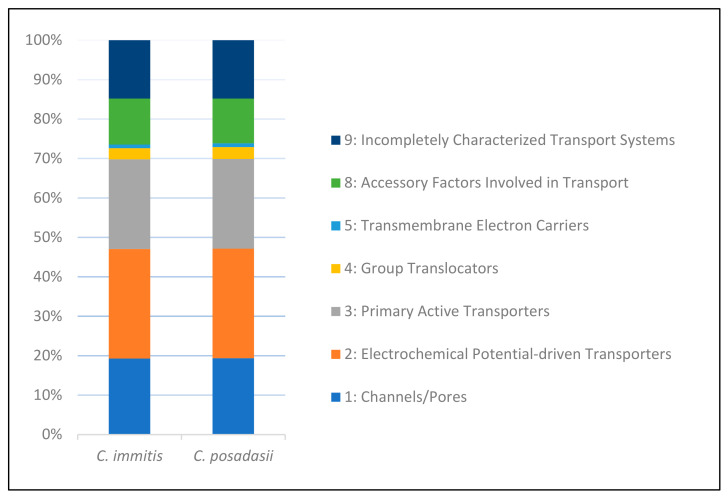
Distribution of transporter types according to the TC system in *Coccidioides* genomes. Class 1: Channels/Pores; Class 2: Electrochemical Potential-driven Transporters; Class 3: Primary Active Transporters; Class 4: Group Translocators; Class 5: Transmembrane Electron Carriers; Class 8: Accessory Factors Involved in Transport; Class 9: Incompletely Characterized Transport Systems.

Combining domain specification and TC nomenclature, we further divided the 1288 and 1235 transporter homologs into seven classes, 25 subclasses, and 269 families in *C. immitis* and *C. posadasii* (Figure 2 and Table 2). The majority of these predicted transporters were not previously characterized.

The most abundant class of transporters is electrochemical potential-driven transporters (Class 2) (Supplementary Table S1). These 358 and 343 secondary carriers account for approximately over 27% of all the transporters in *C. immitis* and *C. posadasii*. The majority of the Class 2 transporters belong to porters (TC Subclass 2.A), consisting of uniporters, symporters, and antiporters [40,41]. The porters in fungal species catalyze the uptake of nutrients such as sugars, amino acids, and efflux of toxic compounds and drugs. Among these porters, the major facilitator superfamily (MFS) transporters have been implicated in multidrug resistance in *Candida albicans* [42,43,44,45], *C. glabrata* [46,47], *C. tropicalis* [48], and *Aspergillus fumigatus* [49]. We identified 140 and 135 MFS transporters in *C. immitis* and *C. posadasii*, respectively. See Section 3.2.2 for detailed analysis.

The second most abundant class is primary active transporters (Class 3) (Supplementary Table S1). They represent about 22% of the transporter repertoire in *C. immitis* and *C. posadasii*. This class of transporters uses a primary source of energy such as ATP to transport solutes across a membrane against their electrochemical gradient. The members of this class are major players in the uptake and excursion of diverse solutes. Most notably, a superfamily of ATP-binding cassette (ABC) transporters are known to confer efflux-mediated antifungal resistance in pathogenic fungi [21]. Our analysis identified 44 and 38 putative ABC transporters in *C. immitis* and *C. posadasii*, respectively. The genomic and phylogenetic analyses of these ABC transporters are seen in Section 3.2.1. In addition, P-type ATPases were found to be present in fungi species such as *A. fumigatus*, *A. nidulans*, *A. oryzae*, *C. neoformans*, *Neurospora crassa*, *Saccharomyces cerevisiae*, and *Schizosaccharomyces pombe* [50]. These active pumps play important roles in ion homeostasis for fungal cell physiology [51]. *C. immitis* and *C. posadasii* genomes contain 20 and 18 P-type ATPases, respectively, including ATPases that translocate calcium, magnesium, copper, phospholipid, and potassium-sodium (Supplementary Table S1).

Class 1 channel/pore proteins are ubiquitous in all living organisms. Over 19% of the transporter homologs in *C. immitis* and *C. posadasii* fall within this class. These transporters facilitate diffusion of solutes in an energy-independent mode. The two largest subclasses in Class 1 present in *Coccidioides* are α-type channels (TC Subclass 1.A) [52], and membrane-bounded channels that form pore complexes (TC Subclass 1.I) [53]. *Coccidioides* possess various cation channels, including calcium, potassium, and transient receptor potential channels with potential roles in cellular signaling and homeostasis. Homologs of these cation channels are present in *S. cerevisiae* [54,55], *C. albicans* [56], *Aspergillus* spp. [57], and *C. neoformans* [58]. Although the detailed physiological function of fungal channels is largely unknown, they are considered as potential drug targets [59]. In addition to channels, *Coccidioides* also possess Nuclear Pore Complex (NPC) transporters (TC family 1.I.1) that bidirectionally transfer macromolecules between the cytoplasm and the nucleus. Besides facilitating nucleocytoplasmic trafficking, the homologs of these porins were shown to be involved in chromatin organization, and gene expression regulation in fungi [53,60,61].

Taking a systems perspective, the TC system also groups various accessory factors that facilitate the transport, but do not directly transport solutes into Class 8. Over 140 predicted homologs fall into Class 8, representing 11% of the total transporter homologs in *C. immitis* and *C. posadasii*. The physiological roles of these putative transporters are largely unknown.

### 3.2. Potential Functionally Important Transporters in C. immitis and C. posadasii

Among the 269 families of predicted transporters, undoubtedly some of the uncharacterized transporters perform important functions in the *Coccidioides* life cycle, for example, the ABC transporter superfamily (TC 3.A.1) and the major facilitator superfamily (MFS) (TC 2.A.1). Examples of potentially important transporters in *Coccidioides* and their homologs in other pathogenic fungi are shown in Table 3.

#### 3.2.1. ABC Transporters

The ABC transporter superfamily is one of the largest protein families that are ubiquitous in all three domains of life [62,63,64,65,66,67]. ABC transporters possess two types of characteristic domains: (1) transmembrane domains, allowing substrate recognition and translocation across membranes, and (2) nucleotide-binding domains, allowing ATP binding and hydrolysis to power the transport process [68,69,70]. ABC transporters are believed to play crucial roles in transferring a wide array of substrates such as sugar, ion, lipid, amino acids, peptides, proteins, etc., for nutrition, signaling, and stress response [63]. Notably, ABC transporters have been widely implicated in the development of multidrug resistance (MDR) to antimicrobials or anticancer drugs [71,72,73]. MDR conferred by ABC transporters is increasingly concerning in fungal pathogens [74,75,76,77,78,79,80,81,82,83,84,85,86]. Comprehensive phylogenetic analyses of 27 other fungal species from 18 orders of five fungal phyla defined a complex evolutionary history and classification of fungal ABC transporters [21]. The ABC system in *Coccidioides*, however, remains to be defined.

**Table 3:** Examples of potentially important transporters in *Coccidioides* and their homologs in other pathogenic fungi.

Transporter TC ^1^	Annotation ^2^	Accession Number (*C. immitis*)	Accession Number (*C. posadasii*)	BLASTP E-Value ^3^	Homologous Sequence in Pathogenic Fungi		
					Gene/ UniProt ID	Annotation/Function	Species [References]
3.A.1.201.10 Multidrug Resistance Exporter (MDR) Family	ABC transporter family protein	XP_001246263.2 XP_001247009.1	XP_003066296.1 XP_003066854.1	0	MDR1 B0Y3B6	ABC multidrug transporter	*Aspergillus fumigatus* [87]
3.A.1.205.6 Pleiotropic Drug Resistance (PDR) Family	ABC transporter	XP_001242727.2	XP_003069892.1	0	AFR1 Q8X0Z3	ABC transporter	*Cryptococcus neoformans* [88]
3.A.1.205.32 Pleiotropic Drug Resistance (PDR) Family	ABC transporter CDR4	XP_001239472.1 XP_001247647.1	XP_003065789.1 XP_003067068.1	0	MDR3 F2SG60	ABC multidrug transporter	*Trichophyton rubrum* [89]
3.A.1.208.41 Drug Conjugate Transporter (DCT) Family	ABC multidrug transporter	XP_001240132.1 XP_004445935.1	XP_003066532.1 XP_003069119.1	0	ECDL K0E4D9	Antifungal Echonocandin B exporter	*Aspergillus fumigatus* [90]
3.A.1.212.3 Mitochondrial Peptide Exporter (MPE) Family	ATP-dependent permease MDL1	XP_001248653.1	XP_003070931.1	0	MDR2 F2RYR3	ABC multidrug transporter	*Trichophyton tonsurans* [91]
2.A.1.2.115 Drug:H^+^ Antiporter-1 (DHA1) Family	Multidrug resistance protein	XP_001240201.2 XP_001247717.2	XP_003065730.1 XP_003069073.1	<2E-59	QDR2 Q6FSQ7	Multidrug transporter	*Candida glabrata* [47,92]
2.A.1.3.83 Drug:H^+^ Antiporter-2 (DHA2) Family	Drug:H^+^ antiporter-2	XP_001241400.2 XP_001241607.2 XP_001247971.2	XP_003065523.1 XP_003070493.1 XP_003070583.1	<4E-35	AFLT Q6UEH3	Efflux pump aflT	*Aspergillus parasiticus* [93]
2.A.1.3.73 Drug:H^+^ Antiporter-2 (DHA2) Family	Major Facilitator Superfamily protein	XP_001240495.1 XP_001241711.1 XP_001241807.1 XP_001242730.2 XP_001243393.1 XP_001245646.2 XP_001246829.2 XP_001247440.1 XP_001247620.2	XP_003065952.1 XP_003066427.1 XP_003066894.1 XP_003068964.1 XP_003069895.1 XP_003070661.1 XP_003071281.1	<4E-21	MFS1 A4ZGP3	Multidrug resistance Mfs1 protein	*Zymoseptoria tritici* [94]
2.A.39.2 Nucleobase: Cation Symporter-1 (NCS1) Family	NCS1 nucleoside transporter	XP_001245665.1	XP_003071266.1	1E-54	FCY21 Q708J7	PURINE-CYTOSINE PERMEASE (PCP)	*Candida albicans* [95]
2.A.40.7 Nucleobase: Cation Symporter-2 (NCS2) Family	Nucleoside transporter	XP_001244274.1	XP_003068546.1	0	AzgA Q7Z8R3	Purine (hypoxanthine/adenine/guanine) transporter	*Aspergillus nidulans* [96]

Note: ^1^ The 5-letter transporter classification ID, in the form of VWXYZ corresponds transporter class, subclass, family, subfamily and the substrate/substrates transported [27]. ^2^ Annotation based on transporter classification. ^3^ The E-value of BLASTP analysis. The lower the E-value, or the closer it is to zero, the more significant the match is.

**Figure 3:**
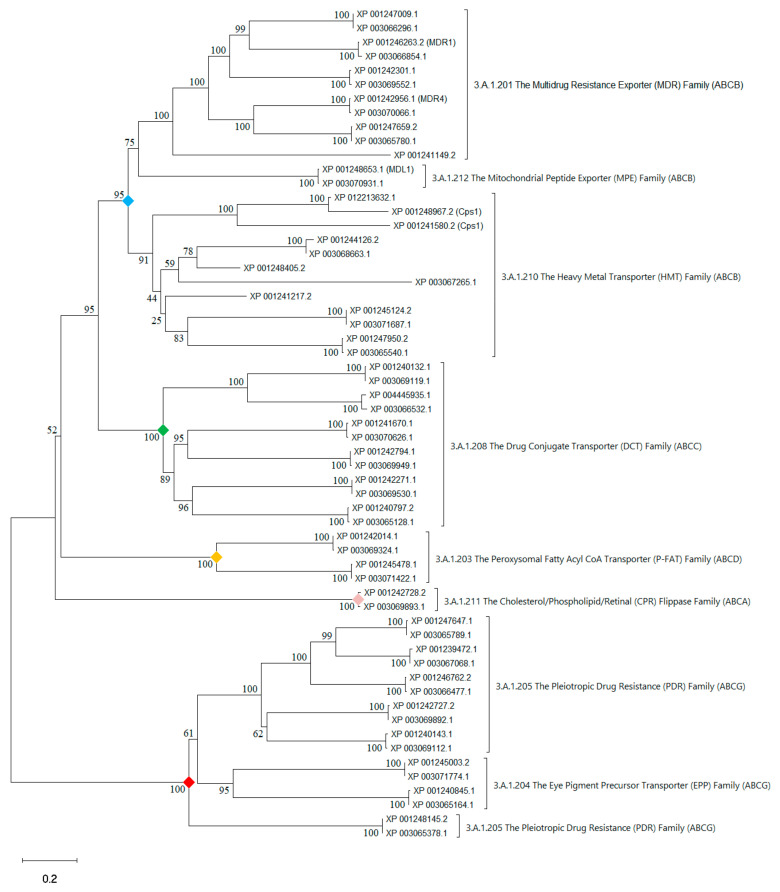
Evolutionary relationships of major ABC transporter families in *C. immitis* and *C. posadasii*. The evolutionary history was inferred using the neighbor-joining method [36]. The percentage of replicate trees in which the associated taxa clustered together in the bootstrap test (1000 replicates) are shown next to the branches [39]. The tree is drawn to scale, with branch lengths in the same units as those of the evolutionary distances used to infer the phylogenetic tree. The evolutionary distances were computed using the Poisson correction method [97] and are in the units of the number of amino acid substitutions per site. Evolutionary analyses were conducted in MEGA11 [38]. Accession numbers: XP_0012XXXXX (*C. immitis* sequences), XP_004445935.1 (*C. immitis*), and XP_0030XXXXX (*C. posadasii* sequences). Each colored diamond corresponds to the branching point leading to a specific ABC group.

Our genomic analyses identified 44 and 38 putative ABC transporters in *C. immitis* and *C. posadasii*, respectively. Phylogenetic analysis revealed that these ABC transporters could be divided into five distinct efflux groups (Figure 3).

ABCB, the largest group, includes three families. The Multidrug Resistance Exporter Family (TC 3.A.1.201) has been widely studied in other fungi species such as *S. cerevisiae*, *S. pombe*, *C. albicans*, *A. fumigatus*, and *C. neoformans* [21]. Four homologs in *C. immitis* and *C. posadasii* are closely related to the *MDR1* gene in *A. fumigatus*, which confers resistance to Cilofungin [87] (Supplementary Table S1). The second member of the ABCB group is the Heavy Metal Transporter (HMT) Family (TC 3.A.1.210). Eight *C. immitis* and three *C. posadasii* HMT homologs are closely related to the vacuolar *HMT1* gene in *S. pombe* [98], which is capable of enhancing heavy metal tolerance in a high calcium content. A mitochondrial *ATM1* gene in *C. posadasii* is homologous to mitochondrial iron transporter *ATM1* in *S. cerevisiae*, which is essential for biogenesis of cytosolic iron/sulfur proteins [99,100,101,102]. The third member of the ABCB group is the Mitochondrial Peptide Exporter (MPE) Family (TC 3.A.1.212). ATP-dependent permease *MDL1* in *C. immitis* and its homolog in *C. posadasii* are evolutionarily related to the *MDR2* gene in *Trichophyton tonsurans* (scalp ringworm fungus), which plays an important role in susceptibility to multiple antifungal drugs [91].

The ABCG group includes two families: (1) The Pleiotropic Drug Resistance (PDR) Family (TC 3.A.1.205). In *C. immitis* and *C. posadasii*, a gene is homologous to the *Afr1* gene, which confers resistance to azole antifungal drugs including fluconazole in *C. neoformans* [88]; two genes are homologous to *MDR3* of *T. tonsurans*, which is an important member of the azole efflux pump network [89]. (2) The Eye Pigment Precursor Transporter (EPP) Family (3.A.1.204), which shows sequence similarity to multidrug/pigment exporter, *Adp1* in *S. cerevisiae* [87].

The ABCC group includes the Drug Conjugate Transporter (DCT) Family (TC 3.A.1.208). The *Coccidioides* sequences in this family are homologous to various drug transporters, for instance, the *YOR1* gene in *S. cerevisiae* conferring resistance to oligomycin, rhodamine B, tetracycline, verapamil, eosin Y and ethidium bromide [103], the *YCF1* gene in *S. cerevisiae* involved in vacuolar metal resistance and drug detoxification [104], the bile acid transporter *BAT1* gene in *S. cerevisiae*, and the *EcdL* gene in *A. fumigatus* conferring resistance to antifungal Echnocandin B [90].

The ABCD group includes the Peroxysomal Fatty Acyl CoA Transporter (P-FAT) Family (TC 3.A.1.203). *C. immitis* and *C. posadasii* each possesses two paralogs of *P-FAT* genes, which may be involved in the fatty acid transport across the peroxisomal membrane [105].

Cholesterol/Phospholipid/Retinal (CPR) Flippase Family (TC 3.A.1.211) is the single member of the ABCA group in *C. immitis* and *C. posadasii*, which may mediate the efflux of cellular cholesterol and phospholipids [106].

#### 3.2.2. Major Facilitator Superfamily (MFS)

MFS (TC 2.A.1) constitutes a large and diverse superfamily of secondary active transporters. Widespread across all three domains of living organisms, MFS transporters move a broad spectrum of small molecules across membranes to maintain important physiological function of cells [107,108,109]. Similar to the ABC superfamily, MFS has been widely recognized in various pharmacological processes by active excursion of cytotoxic compounds [110]. Mounting evidence suggests that MFS transporters are key mediators of antifungal resistance [20,111,112,113,114,115,116]. While an MFS transporter was shown to have undergone fast evolution in the *Coccidioides* lineage, indicating potential significance of MFS in *Coccidioides* adaptation [17], our knowledge of the MFS system in *Coccidioides* remains minimum.

We identified 140 and 135 MFS transporters in *C. immitis* and *C. posadasii*, respectively, representing nearly 11% of the predicted transporters in their genomes. They belonged to 18 families. Of particular interest, abundant members were found in the Drug:H+ Antiporter-1 (DHA1) Family and the DHA2 Family. DHA1 and DHA2 transporters have demonstrated roles in antifungal resistance in fungal species in the genus of *Saccharomyces*, *Candida*, *Cryptococcus* and *Aspergillus* [20,114].

**Figure 4:**
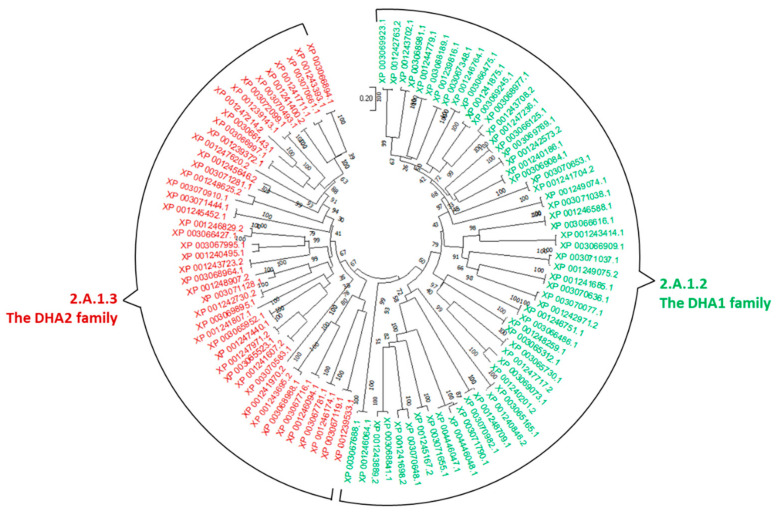
Evolutionary relationships of DHA transporter families in *C. immitis* and *C. posadasii*. The sequences in the DHA1 family are marked in green, and the sequences in the DHA2 family are marked in red. The evolutionary history was inferred using the neighbor-joining method [36]. The percentage of replicate trees in which the associated taxa clustered together in the bootstrap test (1000 replicates) are shown next to the branches [39]. The tree is drawn to scale, with branch lengths in the same units as those of the evolutionary distances used to infer the phylogenetic tree. The evolutionary distances were computed using the Poisson correction method [97] and are in the units of the number of amino acid substitutions per site. Evolutionary analyses were conducted in MEGA11 [38]. Accession numbers: XP_0012XXXXX (*C. immitis* sequences), XP_004445935.1 (*C. immitis*), and XP_0030XXXXX (*C. posadasii* sequences).

Phylogenetic analysis revealed that DHA1 and DHA2 homologs in *Coccidioides* are distributed into two clusters, which is consistent with their distinct structural properties (Figure 4).

The DHA1 cluster (TC 2.A.1.2) includes 29 and 28 members in *C. immitis* and *C. posadasii*, respectively. These genes display sequence homology to DHA1 genes characterized in other fungi (Supplementary Table S1). For example, both *Coccidioides* genomes contain paralogs to the HOL1 gene in *S. cerevisiae*, which is capable of nonselective uptake of histidinol and other cations [117]. Notably, *C. immitis* and *C. posadasii* each possesses two paralogs with high sequence similarity to the QDR2 genes in *Candida* species. QDR2 is known to confer prevalent resistance to a broad spectrum of antifungals including miconazole, clotrimazole, tioconazole, and ketoconazole and quinidine [47,92,118]. One of these two paralogs, XP_001240201.2, appeared to have undergone rapid evolution in *C. immitis* [17].

The DHA2 cluster (TC 2.A.1.3) includes 24 and 21 members in *C. immitis* and *C. posadasii*, respectively. Two paralogs of aflT genes are in the *Coccidioides* genomes; AflT is an efflux pump in the aflatoxin pathway in filamentous fungus *Aspergillus parasiticus* [93]. Four paralogs are present in each of the *Coccidioides* genomes, closely related to the YOR378W gene in *S. cerevisiae*, which is involved in boron stress tolerance [119]. Abundant copies (nine and seven) of genes are present in *C. immitis* and *C. posadasii*, respectively, with high homology to MFS1 genes, which is related to the antifungal resistance in the wheat fungal pathogen *Zymoseptoria tritici* [94].

#### 3.2.3. Other Novel Transporters

Our catalog of *Coccidioides* transporters also includes novel transporters with broad implications in fungal physiology. For example, we found members in the Nucleobase: Cation Symporter-1 (NCS1) Family (TC 2.A.39) and in the Nucleobase/Ascorbate Transporter (NAT) or Nucleobase: Cation Symporter-2 (NCS2) Family (TC 2.A.40) [95,120,121], which may be important components of salvage pathways for purine, pyrimidine, and related metabolites [96]. Two paralogs of TRK genes (TC 2.A.38) were identified in *Coccidioides*. TRK1 and TRK2 were found to participate in potassium uptake and response to internal and external signals in *S. cerevisiae* and *C. albicans* [122,123].

### 3.3. Structural and Biochemical Features of Transporters in C. immitis and C. posadasii

**Figure 5:**
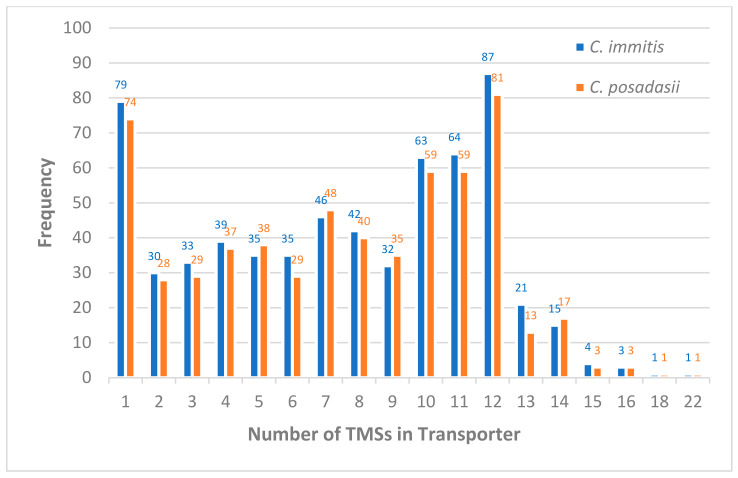
Distribution of transporter topologies in *Coccidioides immitis* and *Coccidioides posadasii* genomes. No TMS helices were identified in 658 predicted transporters in *C. immitis* and 640 predicted transporters in *C. posadasii*.

Transmembrane segments (TMSs) are important structural components for transporters to translocate solutes across membranes. The topology of a transporter is specified by the number of the TMSs, and its overall orientation in the membrane [124]. The number of TMSs is a characteristic feature of fungal transporters; for example, different subfamilies of fungal ABC proteins were shown to possess distinct number of TMSs and nucleotide-binding domains, suggesting structure-function correlations [21,125]. To reveal transmembrane topology of predicted transporters in *Coccidioides*, we performed TMHMM analysis [126]. Similar distributions of TMS topology were shown in two *Coccidioides* genomes. The number of TMSs range from 0 to 22 (Table 4 and Figure 5). While the structural-functional significance of the TMS topology in *Coccidioides* is yet to be elucidated, we found that the majority of membrane transporters are channels/pores, electrochemical potential-driven transporters, group translocators, or electron carriers.

**Figure 6:**
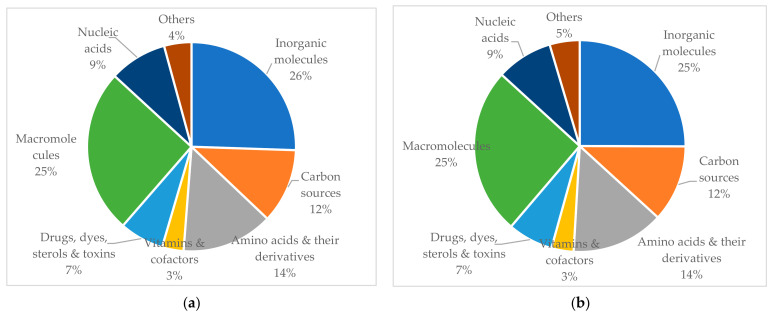
The distribution of substrate types of transporter proteins in *Coccidioide* genomes. The transporters with no identified substrates were excluded. (**a**) Distribution of substrate types in *C. immitis* genome; (**b**) Distribution of substrate types in *C. posadasii* genome. The substrates for predicted *Coccidioides* transporters were predicted based on the Chemical Entities of Biological Interest (ChEBI), an ontology and dictionary focused on small chemical compounds [33].

The putative *Coccidioides* transporters were predicted to be responsible for the translocation of extraordinarily diverse substrates, including inorganic molecules, carbon sources, drugs, toxins, electrons, macromolecules, amino acids and derivatives, nucleic acids, vitamins, and accessory factors (Supplementary Tables S2 and S3). As shown in Figure 6a,b, the two most abundant classes of the substrates in *C. immitis* and *C. posadasii* were inorganic molecules and macromolecules. More specifically, about 200 putative transporters were predicted to use cations and proteins as substrates.

## 4. Discussion

Transporters are essential components for the survival of living organisms. The roles of transporters have been demonstrated in a variety of fungi. Our knowledge of the transport machinery in *Coccidioides*, however, remains limited. The genome annotation laid the groundwork for transporter characterization in *C. immitis* and *C. posadasii* [16,17]. To date, only a small number of transporters in *Coccidioides* have been reported or studied, including MDR1 (a multidrug resistance protein, accession number XP_003069119.1) and PSP1 (a hypothetical lipid transporter, accession number XP_003069236.1) in *C. posadasii* [127], a transmembrane amino acid transporter CIMG_11858 (accession number XP_012214138.1) and a major facilitator superfamily transporter CIMG_09822 (accession number XP_001240201.2) that showed fast evolution in the *Coccidioides* lineage [17], an ABC multidrug transporter CIMG_09753 (accession number XP_001240132.1), and a copper transporter CIMG_10037 (accession number XP_001239015.1) in *C. immitis* [128].

To fill in the critical knowledge gaps in *Coccidioides* biology, here, for the first time, we present a catalog of 1288 and 1235 putative transporters in *C. immitis* and *C. posadasii*, based on exhaustive homology search and comparative genomic analysis. These transporters fall into seven classes, 25 subclasses, and 269 families, with diverse transmembrane topologies and a wide array of substrates. Our hypothesis that *Coccidioides* fungi possess a rich and powerful transport machinery is justified.

It was estimated by the TCDB that transporters constitute about 10% of all cellular proteins [27]. The high content of transporters found in *Coccidioides* is likely an attribute of their adaptation to the complex soil ecosystem and the alien mammalian host system. Soil represents one of most challenging natural environments in which microorganisms scavenge nutrients, produce toxins to competing organisms and resist the effects of such cytotoxic substances. A similarly high content of transporters was observed in soil microbials, especially in the genus of *Streptomyces* [129,130]. However, unlike most Ascomycetes, which are plant pathogens or plant associated, clear evidence suggested that *Coccidioides* have undergone extensive genomic evolution to adapt to the animal host niche [17]. Such adaptation includes at least two major challenges: first, surviving from a plant-associated to an animal-associated nutritional environment, in a desert or a semi-dessert setting, and second, surviving from host immune detection and defense. The distribution of substrates in the *Coccidioides* transporters shows that a large number of transporters use proteins, amino acids and derivatives as substrates (Supplementary Tables S2 and S3), indicative of the need for an animal-associated nutritional niche. Moreover, the observed lineage-specific expansion of transporter families including ABC and MFS may be an outcome of positive natural selection in response to the external stress within a human host and to antifungal treatments, thereby contributing to the development of infectious phenotypes in *Coccidioides* [16,17].

We are bearing in mind that the catalog presented in this study are in silico predictions that await experimental validation. Historically, *Coccidioides* are understudied, partly due to the required biosafety level 3 (BSL3) laboratory containment and the special expertise needed to manage the aerosol risk posed by the large amounts of spores, and the severity of coccidioidomycosis. Experimental assays standard for other organisms can often be time-consuming or cost-prohibitive. Thanks to the advent of the genomic era, the in silico approach is becoming a cost-effective and efficient approach to identify and prioritize genes for wetlab characterization, especially suitable for non-model organisms such as *Coccidioides*.

While the roles of *Coccidioides* transporters in physiology, pathogenesis, and stress response are yet to be fully investigated, this study represents an initial attempt to a systems-level understanding of the mechanisms underlying *Coccidioides* survival and infection. Transporters on the catalog are likely members of the cellular networks associated with nutrient uptake, ion balance, drug excursion, signaling, and regulation. With the availability of high throughput assays, it is possible to integrate various types of omic data to interrogate the expression profiles and associations of transporter genes and their upstream regulators and downstream substrates/effectors in a network perspective [128,131,132,133]. By combining in silico omics-based discovery with wetlab characterization, there is an increased likelihood of identifying new therapeutic targets for these neglected fungal pathogens in the genus of *Coccidioides*.

In addition to providing a catalog of Coccidioides transporters for characterization, this genomic study also raises an important unanswered question: what are the content and diversity of transporters among other pathogenic fungal species? Published work to date mostly focuses on specific transporters or transporter families. For example, Costa et al. [20] surveyed MFS multidrug transporters in pathogenic fungi such as C. albicans, C. tropicalis, C. parapsilosis, C. guilliermondii, C. lusitaniae, C. glabrata, A. fumigatus, and C. neoformans. Kovalchuk and Driessen [21] performed phylogenetic analysis of fungal ABC transporters. Our future direction will be to predict and classify transporters in other pathogenic fungi and conduct comprehensive comparative genomic analyses of the transporters on their diversity and lineage-specific features.

## Figures and Tables

**Table 1:** Distribution of transporters in *Coccidioides*.

Species	Accession Number	Genome Size (Mbp)	# Proteins	# Transporters	% Transporters
*Coccidioides immitis RS*	GCF_000149335.2	28.9	9910	1288	13.0
*Coccidioides posadasii* SOWgp	GCF_000151335.2	27.0	7227	1235	17.1

**Table 2:** Distribution of transporters in each transporter classification (TC) class and subclass in *Coccidioides* genomes. According to the TC system, the first two letters (VW) correspond to the transporter class and the subclass.

Class	Subclass	*C. immitis*	*C. posadasii*
**1: Channels/Pores**		**248**	**239**
	1.A: α-Type Channels	70	67
	1.B: β-Barrel Porins	9	8
	1.C: Pore-Forming Toxins (Proteins and Peptides)	17	14
	1.F: Vesicle Fusion Pores	17	16
	1.H: Paracellular Channels	4	4
	1.I: Membrane-bounded Channels	104	103
	1.N: Cell Fusion Pores	5	5
	1.P: Non-Envelop Virus Penitration Complex: A complex of host cell proteins that allow non-envelop virus to penetrate the endoplasmic reticular membrane.	9	10
	1.Q: Fungal Septal Pores	8	8
	1.R: Membrane Contact Site for Interorganellar Transport	4	3
	1.W: Phage Portal Protein Subclass	1	1
**2: Electrochemical Potential-driven Transporters**		**358**	**343**
	2.A: Porters (uniporters, symporters, antiporters)	353	338
	2.D: Transcompartment Lipid Carrier	5	5
**3: Primary Active Transporters**		**293**	**281**
	3.A: P-P-bond-hydrolysis-driven transporters	244	232
	3.B: Decarboxylation-driven transporters	3	3
	3.D: Oxidoreduction-driven transporters	44	44
	3.E: Light absorption-driven transporters	2	2
**4: Group Translocators**		**36**	**37**
	4.C: Acyl CoA ligase-coupled transporters	23	25
	4.D: Polysaccharide Synthase/Exporters	8	8
	4.E: Vacuolar Polyphosphate Polymerase-catalyzed Group Translocators	2	2
	4.F: Choline/EthanolaminePhosphotransferase 1	3	2
**5: Transmembrane Electron Carriers**		**12**	**12**
	5.B: Transmembrane 1-electron transfer carriers	12	12
**8: Accessory Factors Involved in Transport**		**150**	**140**
	8.A: Auxiliary transport proteins	150	140
**9: Incompletely Characterized Transport Systems**		**191**	**183**
	9.A: Recognized transporters of unknown biochemical mechanism	74	71
	9.B: Putative transport proteins	117	112
**Total**		**1288**	**1235**

**Table 4:** Distribution of topological types of transporters in *Coccidioides* genomes.

# TMSs	*C. immitis*	*C. posadasii*
0	658	640
1	79	74
2	30	28
3	33	29
4	39	37
5	35	38
6	35	29
7	46	48
8	42	40
9	32	35
10	63	59
11	64	59
12	87	81
13	21	13
14	15	17
15	4	3
16	3	3
18	1	1
22	1	1

## Data Availability

The accession numbers of all the sequences are included in the manuscript.

## Supplementary Materials

The following supporting information can be downloaded at: https://www.mdpi.com/article/10.3390/jof8101064/s1, Supplementary Table S1. (A) Putative transporters in *C. immitis*. (B) Putative transporters in *C. posadasii.* Supplementary Table S2. Counts of seven classes of transporter proteins according to the substrate types in *C. immitis*. Transporters without identified substrate(s) were excluded. Supplementary Table S3. Counts of seven classes of transporter proteins according to the substrate types in *C. posadasii.* Transporters without identified substrate(s) were excluded.

## Abbreviations

ABC	ATP-binding cassette
BSL	biosafety level
ChEBI	Chemical Entities of Biological Interest
CPR	Cholesterol/Phospholipid/Retinal
DCT	Drug Conjugate Transporter
DHA	Drug:H+ Antiporter
EPP	Eye Pigment Precursor Transporter
HMT	Heavy Metal Transporter
MDR	multidrug resistance
MFS	major facilitor superfamily
MPE	Mitochondrial Peptide Exporter
NCS	Nucleobase: Cation Symporter
NPC	Nuclear Pore Complex
PDR	Pleiotropic Drug Resistance
PFAT	Peroxysomal Fatty Acyl CoA Transporter
TC	Transport Classification
TCDB	transporter classification database
TMS	transmembrane segment
