# Supplementary material for: Genomic Data Mining Reveals Abundant Uncharacterized Transporters in Coccidioides immitis and Coccidioides posadasii
Source: J Fungi (Basel). 2022 Oct 10;8(10):1064. doi: 10.3390/jof8101064 (PMC9604845; doi:10.3390/jof8101064)
Supplement: Supplementary file 1 [file jof-08-01064-s001.zip › jof-1914855-supplementary/SupplementaryTableS2.pdf]

Supplementary Table S2. Counts of seven classes of transporter proteins according to substrate type in *Coccidioide* i*mmitis*. Transporters without identified substrate were excluded.

**Substrate**	**No. of transporters (class) acting on substrate type**							**Total**
	1. Channels & Pores	2. Primary Carriers	3. Secondary Carriers	4. Group translocators	5. Trans-membrane electron flow carriers	8. Auxiliary proteins	9. (Putative) Poorly characterized	
1. Inorganic molecules (283)								
A. Cation	20	106	77	0	4	0	4	211
B. Anion	2	34	1	4	0	0	0	41
C. electrons	0	3	2	0	4	1	0	10
D. other	15	6	0	0	0	0	0	21
2. Carbon sources (128)								
A. Sugars & polyols	0	38	0	6	0	0	8	52
B. carboxylates	1	48	1	1	0	0	0	51
C. Organoanion	0	2	0	0	0	0	0	2
D. Aromatic compounds	1	18	4	0	0	0	0	23
3. Amino acids & their derivatives (157)								
A. Amino acids	0	72	2	0	0	0	0	74
B. Amines, amides, & organocations	1	33	6	1	0	0	1	42
C. Peptides	0	15	20	0	0	6	0	41
4. Vitamins and cofactors (37)								
A. Vitamins	2	28	0	0	0	0	0	30
B. Cofactors	2	3	0	0	0	1	0	6
C. Siderophores	0	1	0	0	0	0	0	1
5. Drugs, dyes, sterols & toxins (76)								
A. Drugs	0	44	24	0	0	0	0	68
B. Pigments & dyes	0	0	4	0	0	0	0	4
C. Sterols	0	0	2	0	0	0	2	4
6. Macromolecules (282)								
A. Carbohydrates	0	0	0	3	0	0	0	3
B. Proteins	40	1	128	0	0	9	23	201
C. Lipids	4	20	9	19	0	0	15	67
D. other macromolecules	9	2	0	0	0	0	0	11
7. Nucleic acids (99)								
A. Nucleic acids & derivatives	38	33	18	0	0	0	10	99
8. Others (47)								
	27	16	3	0	0	0	1	47
