# Supplementary material for: Genomic Data Mining Reveals Abundant Uncharacterized Transporters in Coccidioides immitis and Coccidioides posadasii
Source: J Fungi (Basel). 2022 Oct 10;8(10):1064. doi: 10.3390/jof8101064 (PMC9604845; doi:10.3390/jof8101064)
Supplement: Supplementary file 1 [file jof-08-01064-s001.zip › jof-1914855-supplementary/SupplementaryTableS3.pdf]

Supplementary Table S3. Counts of seven classes of transporter proteins according to substrate type in *Coccidioide posadasii*. Transporters without identified substrates were excluded.

Substrate	No. of transporters (class) acting on substrate type							Total
	1. Channels & Pores	2. Primary Carriers	3. Secondary Carriers	4. Group translocators	5. Trans-membrane electron flow carriers	8. Auxiliary proteins	9. (Putative) Poorly characterized	
1. Inorganic molecules (267)								
A. Cation	20	97	74	0	4	0	4	199
B. Anion	2	31	1	4	0	0	0	38
C. electrons	0	3	2	0	4	1	0	10
D. other	15	5	0	0	0	0	0	20
2. Carbon sources (125)								
A. Sugars & polyols	0	42	0	6	0	0	4	52
B. carboxylates	1	48	1	1	0	0	0	51
C. Organoanion	0	2	0	0	0	0	0	2
D. Aromatic compounds	1	15	4	0	0	0	0	20
3. Amino acids & their derivatives (151)								
A. Amino acids	0	70	2	0	0	0	0	72
B. Amines, amides, & organocations	1	38	5	1	0	0	1	46
C. Peptides	0	14	13	0	0	6	0	33
4. Vitamins and cofactors (36)								
A. Vitamins	2	28	0	0	0	0	0	30
B. Cofactors	2	3	0	0	0	0	0	5
C. Siderophores	0	1	0	0	0	0	0	1
5. Drugs, dyes, sterols & toxins (74)								
A. Drugs	0	43	24	0	0	0	0	67
B. Pigments & dyes	0	0	4	0	0	0	0	4
C. Sterols	0	0	2	0	0	0	1	3
6. Macromolecules (272)								
A. Carbohydrates	0	0	0	3	0	0	0	3
B. Proteins	38	1	124	0	0	9	23	195
C. Lipids	3	17	9	21	0	0	13	63
D. other macromolecules	9	2	0	0	0	0	0	11
7. Nucleic acids (92)								
A. Nucleic acids & derivatives	37	27	18	0	0	0	10	92
8. Others (49)								
	26	19	3	0	0	0	1	49
